# Managing acute opioid withdrawal with tramadol during COVID-19 lockdown in a peri-urban setting

**DOI:** 10.4102/phcfm.v14i1.3386

**Published:** 2022-09-28

**Authors:** Urvisha Bhoora, Natasha R. Gloeck, Andrew Scheibe

**Affiliations:** 1 Department of Family Medicine, Faculty of Health Sciences, University of Pretoria, Pretoria, South Africa

**Keywords:** tramadol, heroin, *nyaope*, opioid dependence, withdrawal management, detoxification, South Africa

## Abstract

**Background:**

The coronavirus disease 2019 (COVID-19) has highlighted the scope of heroin dependence and need for evidence-based treatment amongst marginalised people in South Africa. Acute opioid withdrawal management without maintenance therapy carries risks of increased morbidity and mortality. Due to the high costs of methadone, Tshwane’s Community Oriented Substance Use Programme (COSUP) used tramadol for opioid withdrawal management during the initial COVID-19 response.

**Aim:**

To describe demographics, route of heroin administration and medication-related experiences amongst people accessing tramadol for treatment of opioid withdrawal.

**Setting:**

Three community-based COSUP sites in Mamelodi (Tshwane, South Africa).

**Methods:**

A retrospective cross-sectional study was conducted. Data were collected using an interviewer-administered paper-based tool between April and August 2020. Descriptive statistics were used to analyse data.

**Results:**

Of the 220 service users initiated onto tramadol, almost half (*n* = 104, 47%) were not contactable. Fifty-eight (26%) people participated, amongst whom most were male (*n* = 55, 95%). Participants’ median age was 32 years. Most participants injected heroin (*n* = 36, 62.1%). Most participants experienced at least one side effect (*n* = 47, 81%) with 37 (64%) experiencing two or more side effects from tramadol. Insomnia occurred most frequently (*n* = 26, 45%). One person without a history of seizures experienced a seizure. Opioid withdrawal symptoms were experienced by 54 participants (93%) whilst taking tramadol. Over half (*n* = 38, 66%) reported using less heroin whilst on tramadol.

**Conclusion:**

Tramadol reduced heroin use but was associated with withdrawal symptoms and unfavourable side effects. Findings point to the limitations of tramadol as opioid withdrawal management to retain people in care and the importance of access to first-line opioid agonists.

**Contribution:**

This research contributes to the limited data around short-acting tramadol for opioid withdrawal management in the African context, with specific focus on the need for increased access to opioid agonists for those who need them, in primary care settings.

## Introduction

### Opioid use context

Globally, around 58 million people used opioids at least once in 2018.^1^ Opioids are ‘compounds that are extracted from the poppy seed as well as semisynthetic and synthetic compounds with similar properties that can interact with opioid receptors in the brain’.^1^ Opioid use accounted for 66% of the estimated 167 000 deaths related to drug use disorders in 2017.^2^ Most people dependent on opioids used heroin, which is also the most widely injected opioid.^1^ More than 11 million people injected drugs globally in 2018.^2^ People who inject drugs (PWIDs) are at higher risk of exposure to human immunodeficiency virus (HIV) and viral hepatitis C (HCV) and overdose than people who use drugs through other modes of administration and in comparison to the general population.^3^

In South Africa, available data point to a rising prevalence of illicit opioid use in the past two decades.^4^ Findings from a recent (2020) study suggest that 400 000 people in South Africa use heroin daily and that there are an estimated 82 500 PWID.^5^

### Detoxification, opioid substitution therapy and the South African context

Detoxification is the process of managing acute opioid withdrawal by administering opioid substitution therapy (OST) and then reducing the dose in a tapered down regimen. Detoxification is not the recommended treatment for opioid dependence as it is associated with higher levels of return to illicit opioid use, as well as increased mortality and morbidity when compared to OST.^6^ Methadone is the most widely used opioid agonist medication for detoxification and OST.^6^ Potential harms associated with opioid use can be further reduced through needle and syringe programmes for people who inject opioids, naloxone distribution, psychosocial support, community outreach and the prevention, diagnosis and treatment of communicable diseases, including HIV and HCV as part of integrated approaches.^7^

The pharmacological treatment of opioid dependence in South Africa is limited.^8^ Methadone is listed on the South African Adult Hospital-Level Essential Medicines List (EML) and Standard Treatment Guidelines (STGs) for in-patient acute opioid withdrawal (detoxification) using a tapering regimen over 3 to 14 days.^9^ However, methadone access in the public sector is limited to a few hospitals and detoxification centres, mostly in metropolitan areas. The limited methadone access is partly due to the high cost of methadone, as well as partly due to local resistance to pharmacological treatment of opioid dependence, through policies that promote abstinence-only treatment goals or do not support OST maintenance therapy.^10^ Methadone in South Africa costs between R103.43 and R173.26 per day (around $7 – $12) for the lowest suggested maintenance dose of 60 mg, which is 50 times higher than in other middle-income countries like Kenya.^11,12^ Potential reasons for the elevated cost include limited competition due to a single supplier, limited prescribing volumes as methadone is not on the EML for maintenance therapy and not on the prescribed minimum benefits package of medical aids and limited capacity of clinicians to confidently manage opioid use disorders with pharmacological interventions.^10^ In 2020, only 1% of the country’s PWIDs had access to OST, almost exclusively through the not-for-profit and private sectors.^13^

Tramadol is listed on the hospital-level STG as the second-line agent for in-patient acute opioid withdrawal.^9^ Tramadol is a widely available and affordable opioid analgesic with a short half-life of 6–7 h. The STG recommends 100 mg tramadol every 4 h for analgesia (600 mg per day) and 200 mg every 12 h for 14 days for opioid detoxification.^9^ The manufacturer’s recommended maximum daily dose is 400 mg.^14^ Common side effects (defined as occurring in more than 1% of people who take tramadol) include dizziness, nausea, vomiting, sweating and dry mouth. Seizures are noted as a potential uncommon side effect (defined as occurring in less than 1% of people who take tramadol).^14^

Several studies have assessed the use of tramadol to manage acute opioid withdrawal.^15,16,17^ One study conducted in 2012 in Iran proposed that tramadol 600 mg/day (given as 200 mg 8 h) used as detoxification may be as effective as methadone (60 mg/day) in the control of withdrawal and could be considered as a potential substitute for methadone.^18^ Another study conducted at the Behavioural Research Unit at Johns Hopkins University in 2007 found that oral tramadol doses of 200 mg and 400 mg daily could suppress opioid withdrawal in mild to moderate opioid dependence, though the suppression was preceded by transient and dose-related incidence of unpleasant side effects.^19^ A systematic review of randomised controlled trials also showed the value in tramadol in managing mild to moderate withdrawal cases but at higher doses than prescribed for analgesia.^15^ Evidence for the use of tramadol in cases of severe opioid withdrawal is limited.^15^

### Responding to opioid dependence in the City of Tshwane

Accurate estimates of the number of people who use heroin in the City of Tshwane do not exist. Heroin is commonly mixed with bulking agents (e.g. caffeine, inactive powders or other pharmaceutical opioids) and known locally as *nyaope*.^20^ Heroin is also the most widely injected drug in the city. In 2017, a population size estimation linked to a respondent-driven sampling survey amongst PWIDs estimated the population of PWIDs to be 4514.^21^ More recent programmatic data reflect that between January and June 2020, 43 030 PWIDs accessed harm reduction services in the city.^22^ Between July and December 2020, 6154 PWIDs were reached with harm reduction services in the city.^23^ Because of the growing use of heroin and the potential harms associated with heroin use, the City of Tshwane adopted an evidence-based harm reduction approach. As part of this change, the University of Pretoria’s Department of Family Medicine implemented the Community Oriented Substance Use Programme (COSUP). The COSUP is the first publicly funded, community-based programmatic response to drug use in South Africa, and it is funded by the City of Tshwane.^24^ Since inception in 2016, COSUP established 20 service delivery sites across the city. Since then, a few sites have been combined to save resources, and by 2020, there were 17 active sites. In 2017, the first COSUP site in Mamelodi, an urban township (underdeveloped, residential areas that during apartheid were reserved for nonwhite people) in the east of Tshwane, was established to provide OST (using methadone) and related services.

As methadone is not on government tender for OST maintenance but only as part of acute withdrawal management within hospital settings, COSUP procures it from private pharmacies. The high cost of methadone limits the number of people who can be treated. At the end of March 2020, 281 people had been initiated on OST across the three Mamelodi COSUP sites.^25^

### Opioid dependence during the COVID-19 pandemic

COVID-19 was declared a national disaster in South Africa on 15 March 2020. A five-level COVID-19 alert system (level 5 being a time of high COVID-19 spread where drastic measures are needed to contain the spread and level 1 being a time of low COVID-19 spread where most normal activity can continue) was implemented to curb the spread of the infection.^26^ South Africa went into alert level 5 from 26 March until 30 April 2020, which confined people to their place of residence unless performing an essential service, obtaining essential goods or services, or seeking medical attention.^26^ The lockdown also necessitated the state to create temporary shelters for the homeless.^27^ The restriction of movement and placement of homeless people into shelters – initially a single mass shelter (± 2000 people) and later smaller shelters of varying capacities – unmasked the unmet need for opioid dependence treatment in Tshwane.^27^ The gradual easing of lockdown levels, which allowed people to move more freely and generate an income, occurred between May and December 2020.

A total of 1189 homeless reported current opioid use as part of their admission process in 25 shelters in the initial COVID-19 response in Tshwane.^28^ Across the 17 COSUP sites, there were 669 people on OST at the end of March 2020, with more people presenting for assistance with acute opioid withdrawal as the lockdown progressed. The demand for OST exceeded supply. The COSUP programme requested medications from Pharmaceutical Services at Tshwane Health District. Personal communication between COSUP’s head pharmacist and clinical head confirms that pharmaceutical services were able to allocate 19 860 mL methadone (sufficient for 15 mL [30 mg] daily for 362 people for 4 days) and provide short-acting 50 mg tramadol capsules as needed to COSUP to aid in the management of acute opioid withdrawal at its sites and the shelters. As the need for opioid withdrawal management steadily increased, and no additional methadone was available, COSUP management decided to use tramadol at COSUP sites and shelters. Tramadol was easily accessible and in stock at the Tshwane Health District. The management protocol included taking a history, including a baseline assessment of opioid use based on the World Health Organization (WHO) Alcohol, Smoking and Substance Involvement Screening Test (ASSIST) score.^29^ A score of 27 or more is considered high risk with important health consequences. Clients were also examined, assessed for opioid withdrawal, counselled and then prescribed tramadol. Supervising doctors prescribed tramadol and the medication was administered by clinical associates (mid-level clinicians). Tramadol dosing started with 200 mg 12 hourly, increasing to a daily maximum of 600 mg (200 mg 8 hourly), depending on tolerability and effect.

## Aims and objectives

This study describes the demographics, comorbidities and baseline heroin use characteristics as well as craving management, side effects and persistence in opioid dependence management amongst people accessing tramadol from April to August 2020 at COSUP sites in Mamelodi.

## Research methods and design

### Study design

Retrospective cross-sectional descriptive study.

### Study setting

The study took place at the three COSUP Mamelodi sites, which are within a 10-km radius from each other: Ikageng Holy Bible Church (established in early 2017); Mamelodi Lusaka (established in mid-2017) and Mamelodi Regional Hospital (established in 2019). Mamelodi has a population of just under 335 000 people.^30^ The *Group Areas Act* under the apartheid system designated Mamelodi an area for black people only, and there have not been significant changes in the demographics to date.^28^ The population is mostly middle to low income, with just under 20% of the population being unemployed and only 0.6% of the population earning over R610 000 ($45 000) per annum.^30^

### Study population and sampling strategy

The population included people aged 18 years and older who were started on tramadol for the management of opioid withdrawal between April and August 2020 at the three sites. People on OST prior to April 2020 and those who were uncontactable, uninterested or did not consent were excluded from the study.

Between September and December 2020, COSUP staff members attempted to contact all clients who were started on tramadol. Clients were informed about the study and offered to participate when they visited the site or when they were seen in the community by COSUP staff members during outreach. Study team members also attempted to contact clients telephonically.

### Data collection

Data were collected in person by a trained clinical associate based at the respective site using a paper-based data collection tool and client file records.

The study tool collected information on demographics (age, sex), baseline substance use history (opioid ASSIST score, method of heroin use, age of first use, age of first injecting, amount of heroin used per day), past medical history (chronic illnesses and medication, including history around HIV, tuberculosis, hepatitis B and C, epilepsy, diabetes, asthma, hypertension and any mental health diagnoses) and information related to the use of tramadol for opioid withdrawal management (initiation date, termination date, duration on tramadol, initiation and final dose, reasons for termination and tramadol side effects) and opioid craving, withdrawal symptoms whilst on tramadol, previous OST and interest in long-term OST. Information related to the use of tramadol was determined by the participants’ experience.

Elicited side effects were taken from the tramadol package insert and the literature.^9,14,31^ Side effects were graded as mild (symptoms causing no or minimal interference with usual activities with intervention not indicated), moderate (symptoms causing greater than minimal interference with usual activities with intervention indicated), severe (symptoms causing inability to perform usual activities with intervention or hospitalisation indicated) and potentially life-threatening.^32^ Participants were asked to grade each side effect they experienced. Participants were asked about the timing of the side effect in relation to their tramadol dose (< 30 min, 30 min – 2 h, 2–6 h, > 6 h) and resolution with time (did not resolve, resolved within 1 week, resolved between 1 and 2 weeks, resolved after 2–4 weeks and resolved after 4 weeks).

Opioid withdrawal symptoms were determined using signs and symptoms from validated opioid withdrawal scales.^6^ Participants were asked about the timing of the withdrawal symptoms in relation to their tramadol dose (< 30 min, 30 min – 2 h, 2–6 h, > 6 h) and if they resolved with time (did not resolve, resolved within 1 week, resolved between 1 and 2 weeks, resolved after 2–4 weeks and resolved after 4 weeks).

### Data analysis

Data were cleaned in Microsoft Excel and then imported into Stata 15 (Statacorp., USA) for analysis. Descriptive statistics (mean, median, proportion, standard deviation [s.d.] and interquartile range [IQR]) were used for numerical variables to describe the sample and reported experience of tramadol.

### Ethical considerations

This study forms part of the emergency research conducted by COSUP during the COVID-19 pandemic. Approval was granted by the Health Sciences Research Ethics Committee (reference number 310/2020) of the University of Pretoria in the Gauteng province of South Africa. Participants gave written informed consent before taking part in the research. There was no reimbursement for participation. Community Oriented Substance Use Programme staff receive training around the provision of sensitive and appropriate care to people who use drugs. Community Oriented Substance Use Programme clients are not required to disclose personal information to access services. No names were captured on the data collection tools used for this study.

## Results

**FIGURE 1:**
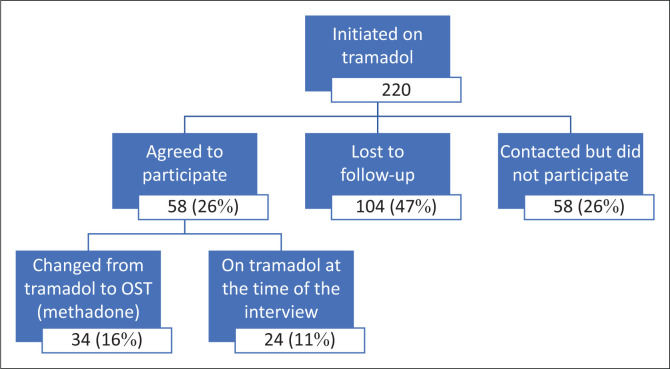
Breakdown of service users initiated onto tramadol and those who were noncontactable, declined or consented to participate in the study.

During the study period, as per Figure 1, 220 service users were initiated onto tramadol. Of those, 58 (26%) participated in this research, 58 (26%) were contacted but chose not to participate and 104 (47%) were not contactable.

Participants were initiated onto tramadol during levels 5, 4 and 3 of the national COVID-19 lockdown, with the majority initiated during level 5 (*n* = 35, 60%) (27 March to 30 April 2020), followed by level 4 (*n* = 14, 24%) (01 to 31 May 2020) and level 3 (*n* = 9, 16%) (01 June to 17 August 2020).^26^ Participants had a median age of 32 years and were mostly male (95%), and the majority injected heroin (62%). The median baseline ASSIST score for opioid use was 33, with a median age of first use of heroin at 20 years and of first injecting of any drug at 27 years. The median length of heroin use was 11 years (IQR 7–15 years). Participants used a median of five bags of heroin per day prior to using tramadol, equating to approximately 1.25 g of heroin daily. Further details can be seen in Table 1.

**TABLE 1:** Demographics and drug use practices of study participants (*n* = 58).

Participant characteristics	Mamelodi Regional Hospital (*n* = 24)				Ikageng (*n* = 17)				Lusaka (*n* = 17)				Total (*n* = 58)			
	*n*	%	median	IQR	*n*	%	median	IQR	*n*	%	median	IQR	*n*	%	median	IQR
Male	24	100	-	-	16	94	-	-	15	88	-	-	55	95	-	-
Female	0	0	-	-	1	6	-	-	2	12	-	-	3	5	-	-
Age (years)	-	-	32	28–36.5	-	-	31	27.5–33.5	-	-	30	26.5–34	-	-	31.5	27.8–34.3
Baseline opioid ASSIST score	-	-	33	31–36.5	-	-	36	31–39	-	-	33	30–33	-	-	33	31–36
Main method of use – smoking	8	33	-	-	4	24	-	-	10	59	-	-	22	38	-	-
Main method of use – injecting	16	67	-	-	13	77	-	-	7	41	-	-	36	62	-	-
Age of first heroin use	-	-	21	18–26	-	-	17	15.5–21.5	-	-	21	16.5–23.5	-	-	19.5	17–24
Age of first injecting use (*n* = 36)	-	-	28.5	21–31.5	-	-	28	24.5–29.5	-	-	24	21–27	-	-	27	22–30
Number of heroin bags used daily	-	-	5	4–6	-	-	5	4–6	-	-	5	4–7.5	-	-	5	4–6

IQR, interquartile range; ASSIST, Alcohol, Smoking and Substance Involvement Screening Test.

Twenty-one (29%) participants reported having a known chronic disease – 16 were HIV positive (of those, nine were on antiretroviral therapy [ART]) and nine had other chronic health diagnoses which included tuberculosis, HBV, HCV, diabetes, asthma, hypertension, epilepsy and mental health diagnoses. A summary of self-reported chronic disease can be seen in Table 2. Four of these participants had more than one chronic disease (one with HIV and asthma, one with HIV and schizophrenia, one with epilepsy and depression and one with epilepsy and schizophrenia).

**TABLE 2:** Self-reported chronic diseases (*n* = 58).

Chronic disease	Mamelodi Regional Hospital (*n* = 24)		Ikageng (*n* = 17)		Lusaka (*n* = 17)		Total (*n* = 58)	
	*n*	%	*n*	%	*n*	%	*n*	%
**HIV positive (all)**								
**Total (*n* = 58)**	8	14	5	9	3	5	16	28
PWID (*n* = 36)	6	17	5	14	3	8	14	39
PWUD (*n* = 22)	2	9	0	0	0	0	2	9
**HIV positive and on antiretroviral therapy**								
Total (*n* = 16)	5	31	4	25	0	0	9	56
PWID (*n* = 14)	3	21	4	29	0	0	7	50
PWUD (*n* = 2)	2	100	0	0	0	0	2	100
**Other chronic conditions** †								
Total	4	17	2	12	3	18	9	16

† , includes tuberculosis, diabetes, asthma, hypertension, epilepsy and mental health diagnoses – no participants self-reported being hepatitis B and/or C positive.

PWUD, people who use drugs (those who do not inject heroin); PWIDs, people who inject drugs.

Twenty-three (40%) participants had previously had an opioid agonist or partial agonist prescribed to them by a doctor, amongst whom 22 had previously used methadone. The median number of days receiving an agonist or partial agonist was 42 (IQR 21–76.5). Six of those previously on an opioid agonist or partial agonist were known to COSUP.

For all participants, the total daily dose of tramadol at initiation was 400 mg. The median final daily dose of tramadol was 500 mg (IQR 400 mg – 600 mg). The median number of days on tramadol was 55 (IQR 24–86).

Reasons for termination of tramadol included that they were moved to OST maintenance (*n* = 33, 57%), tramadol did not work (*n* = 13, 23%), preferred to continue with heroin use (*n* = 4, 7%), unspecified reasons (*n* = 4, 7%) and intolerable side effects of tramadol (*n* = 2, 4%).

Most participants (*n* = 47, 81%) experienced at least one side effect associated with their tramadol use, with 37 (64%) experiencing two or more side effects. The most common side effect was insomnia (45%), followed by dizziness (41%), nausea (31%) and sweating (29%).

**FIGURE 2:**
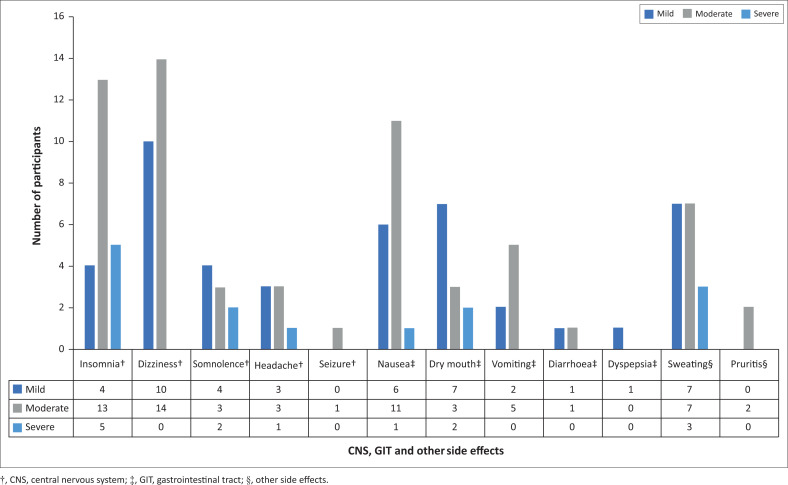
Number of participants experiencing mild, moderate and severe side effects.

Twelve participants reported experiencing at least one side effect that they deemed to be severe. Two of the 12 reported experiencing two side effects that they reported as being severe. Five participants experienced at least one moderate and one severe side effect. The frequency, type and grade of side effects as reported by participants are in Figure 2.

In terms of severe central nervous system (CNS) side effects, five participants reported experiencing severe insomnia, four of whom struggled to fall asleep and one who kept waking up. In all five, the insomnia developed in the first week and did not resolve over time. Two participants complained of severe somnolence which developed within 30 min – 2 h of taking tramadol. For one participant this did not resolve, and for the other, it resolved in less than 1 week. One participant complained of a severe headache which developed within 30 min – 2 h of taking tramadol, after taking tramadol for 1–2 weeks. The headache resolved within 1 week.

In terms of severe gastrointestinal tract side effects, one participant reported severe nausea which developed in the first week, 30 min to 2 h after taking tramadol. The nausea did not resolve over time. Two participants complained of a severely dry mouth. Both developed the symptom after taking tramadol, resolving within the first week in one person and persisting for 2–6 h after taking tramadol in the other.

Three participants reported experiencing severe sweating, which started and resolved during the first week of taking tramadol.

One participant reported a seizure whilst taking tramadol, which they graded as ‘moderate’. The participant was not hospitalised and the seizure self-abated. The seizure occurred more than 1 month after starting tramadol and did not seem to be associated with the timing of when tramadol was taken.

Most participants (*n* = 54, 93%) experienced opioid withdrawal symptoms whilst taking tramadol. The majority (*n* = 48, 83%) experienced two or more withdrawal symptoms. The most common withdrawal symptoms were yawning (*n* = 47, 81%), sweating (*n* = 35, 60%) and general body pain (*n* = 30, 52%). Excessive lacrimation and abdominal cramps were also common (50% respectively). Other withdrawal symptoms experienced included rhinorrhoea, restlessness and gooseflesh. The majority of participants reported experiencing opioid cravings (*n* = 48, 83%) whilst on tramadol, with just under a quarter of all participants reporting severe cravings (*n* = 14, 24%). For six participants (11%), there was no alleviation of craving whilst on tramadol. Nine participants (16%) reported relief for 30 min or less, 17 (30%) reported relief for 30 min to 2 h, 15 (26%) participants reported relief lasting between 2 and 6 h, whilst 10 participants (18%) could not recall the length of relief from cravings whilst using tramadol.

Of the participants, 32 (55%) reported concurrent heroin use whilst on tramadol, with the majority (*n* = 18, 56%) using at least two times a week. However, most participants (*n* = 38, 66%) reported using less heroin whilst on tramadol compared to before being on tramadol. The statistical significance of the decrease in use could not be assessed. Twenty-five (43%) participants reported concurrent use of other substances whilst on tramadol; of those 25, 14 (56%) used cannabis, seven (28%) used cocaine and three (12%) used alcohol and methamphetamine, respectively.

When asked if they were interested in joining a long-term OST maintenance programme to manage heroin (*nyaope*) use, all 58 participants indicated their wish to join such a programme.

## Discussion

Heroin use is not a new phenomenon to South Africa, or to the City of Tshwane. However, during lockdown, there was a 78% increase in the total number of clients seeking management for acute opioid withdrawal across the Mamelodi COSUP sites. This increase speaks to a likely notable opioid dependence in this community, which was negatively affected by COVID-19. Participant initiation dates show a relationship with the levels of lockdown, where just under two-thirds of participants were initiated at level 5 of the lockdown alert response. Many people who use drugs generate income through informal street-based trades such as car guarding or begging.^33^ During level 5, when all South Africans were forced to remain home, few people with opioid dependence could generate an income, and therefore few had money to buy heroin. Similarly, a Norwegian study showed that during the early phase of the pandemic in Norway, drug availability was reduced and drug prices increased.^34^ Published African or South African data on this topic are limited, and although there are considerable differences between Norway and South Africa, the effect of COVID-19 was felt worldwide with similarities in countries that are vastly different.

Of those participants who had previously been prescribed OST (opioid agonist or partial agonist), few had previously accessed COSUP services. Outside of COSUP, people in Tshwane have to fund OST themselves. Although people with prior exposure to methadone or buprenorphine had it for short duration, those who were part of COSUP would have had the option to continue with maintenance therapy. Outside of COSUP, few people are able to afford the cost of agonist therapy as maintenance. As a result, these people then receive longer term withdrawal management. Self-funding OST in Tshwane has also been shown to be negatively associated with retention in care.^35^ It is likely that many people who use drugs in Mamelodi were aware of COSUP, as people are well networked and the service had been operational for the four years before COVID-19. It is possible that people were either not ready or did not want to initiate OST before lockdown and only sought help once faced with the interruption to income generation related to lockdown and a disruption in their access to heroin.

About one-third of participants were self-reported to be HIV positive, most of whom injected drugs. The Global AIDS Strategy 2021–2026 states that, in addition to the 95-95-95 testing and treatment targets within all subpopulations and age groups, 90% of PWIDs should have access to comprehensive harm reduction services integrating or linking to hepatitis C, HIV and mental health services.^3^ We can see that in this cohort, only half of those PWIDs who were HIV positive reported being on ART. There may be various reasons for this, but one of the most damaging may be that many substance users face stigma at healthcare facilities. Stigma is a global issue and Nyblade et al. state, ‘[*s*]tigma in health facilities undermines diagnosis, treatment, and successful health outcomes’.^36^ Harm reduction is still not a readily accepted practice in South Africa, despite its promotion through existing policies.^37^

Hepatitis B and C testing does not yet form part of the workup for substance users joining COSUP. This is in part due to resource constraints. Whilst hepatitis B testing forms part of the National Department of Health’s protocol for the workup of HIV positive persons, hepatitis C testing does not. None of this cohort self-reported to be hepatitis B and/or C positive. Given the number of injecting drug users in this cohort, it is unlikely that none of the participants had hepatitis B and/or C. A study published in 2019 showed the prevalence of hepatitis B and C amongst PWIDs in Tshwane to be between 5% – 7% and 79% – 85%, respectively.^38^

Tramadol side effects were experienced by over 80% of participants, with just under 65% experiencing two or more side effects. Our study found insomnia and dizziness, both CNS effects, to be the most commonly reported side effects, with most participants grading them as moderate.

Interestingly, insomnia is not listed as a common side effect in the tramadol package insert.^14^ Substance use can impact sleep patterns during active use, and in acute withdrawal, insomnia is one of the most common complaints amongst patients with a substance use disorder.^39^ Insomnia is not used as a measure of withdrawal in validated assessment scales (e.g. clinical opioid withdrawal scale).^40^ Whether the insomnia reported was linked to tramadol or a symptom of opioid withdrawal is unclear, but its effect on participants and the need to manage it appear to be important.^39^

Tramadol may increase the risk of seizures as it lowers the seizure threshold.^9,14^ As such, tramadol should be used with caution in persons with a history of seizures or epilepsy. Neither of the two participants with epilepsy reported seizures; however, one participant had their first seizure whilst on tramadol, which did not recur after tramadol was stopped. Unlike several of the other side effects graded by participants as severe and affected their well-being, seizures carry notable morbidity and can affect neurological functioning. Seizures can have major implications resulting from falls and injuries and can also have negative psychosocial consequences that develop.^41^ As such, medication that can result in seizures should be prescribed with caution and patients warned to report any possible resulting seizures to a medical doctor.^14^

More than 50% of the participants used heroin whilst on tramadol. This could be explained by many factors. The majority of participants reported still experiencing opioid withdrawal symptoms and cravings after taking tramadol. Whilst cravings are not recognised as physical withdrawal symptoms, they are recognised as psychological symptoms.^42^ Cravings can have negative emotional effects and can also have serious psychiatric manifestations, such as suicidal ideation and depressed mood, and their management is therefore an important treatment target.^42^ With short-acting tramadol capsules, onset of action generally occurs within 1 h and peaks within 2–3 h after administration, with the recommended dosing interval being 4–6 h.^14^ There is some evidence that shows that extended release formulations of tramadol are acceptable to manage opioid withdrawal symptoms.^16^ Long-acting formulations are, however, not readily available at primary health care level and were not evaluated as part of this study.

The side effects, limited management of withdrawal symptoms and cravings together with the high pill burden (up to twelve 50 mg capsules per day) may have contributed to participants being unwilling to up-titrate to the maximum dose of 600 mg/day. It could also explain why more than half of the participants used heroin concurrently with their tramadol. The data only reflect the dosage provided to participants. Data were not collected on whether or not clients or participants were unwilling or unable to increase their dosage. Despite these challenges, just under two-thirds of participants reported a decrease in their daily heroin use. This could be due to a combination of factors, including the partial effect of tramadol in managing withdrawal symptoms or the financial constraints encountered as a result of the lockdown.

### Limitations

Only 26% of people placed on tramadol in these sites participated in the study. Just under half (47%) of people started on tramadol were lost to follow-up, and a further 26% were contacted but chose not to participate in this study. Some of these people may have returned to using heroin, and those who were not contactable and those who declined to participate could have had different experiences of tramadol than those who did participate. People who were contactable and consented to participate were all interested in joining a maintenance programme, which suggests a selection bias. These people appear to be motivated to access treatment for their heroin use.

Furthermore, the findings cannot be generalised to the broader population because of the small sample size and because no random sampling was used.

Only two of the 58 participants were on tramadol at the time of the interview. Tramadol was used for withdrawal management in a crisis situation. The magnitude of the need for opioid dependence management in Mamelodi at the time was not anticipated. As the situation unfolded, the importance of studying the use of tramadol became clearer and the tools were developed, delaying data collection and resulting in probable recall bias. Furthermore, given that the number of participants who stopped due to tramadol not working exceeded the number who stopped due to side effects, it would have been helpful to further understand the concept of ‘did not work’, but this information was not collected. Although validated withdrawal scales were used, there was likely some overlap between the symptoms of withdrawal and the side effects of the medication, which could have been explored further.

As side effect grading was recorded as a subjective experience by the participant, some side effects (sweating, dry mouth) were graded higher (e.g. severe) than a clinician would have graded them. It is important, however, that their experience be documented, as these side effects and the experiences thereof would likely affect adherence to treatment and follow-up.

Women and the challenges experienced by women were not explored in this study. Additional insights are needed to understand how to better engage and retain women who use opioids into health services.

## Conclusions

This study provides additional evidence to highlight the need for access to evidence-based services for people with opioid dependence. The findings point to short-term tramadol’s partial effect in moderating acute opioid withdrawal symptoms and reducing heroin use. However, the frequency and nature of side effects coupled with the limited effect on cravings suggests the inadequacy of short-acting tramadol to manage acute opioid withdrawal. In addition, using tramadol for epileptic patients should be considered very carefully and these patients should ideally be prioritised to receive methadone or buprenorphine.

With the high cost of methadone in South Africa and tramadol being listed as a second-line drug on the EML, it is essential that the use of short-acting tramadol for acute opioid withdrawal be carefully examined. Further research comparing the use of long-acting tramadol and other agonist medications in the management of opioid withdrawal is warranted. The overlap of withdrawal symptoms and side effects of medication should also be closely evaluated. People placed on tramadol could be monitored as part of cohort studies, but we would recommend focus not be diverted away from the use of recommended opioid agonist and partial agonist therapies.

The findings have also highlighted the burden of HIV amongst people who use and inject opioids in the city and the need to support them to access care. Increased access is needed for comprehensive and integrated harm reduction services, including needle and syringe services and long-term OST to prevent new infections.

The interest that participants expressed to access long-term OST demonstrates an unmet need. Efforts should continue towards making long-term OST more accessible. For this to happen, policy supporting OST needs to be adequately funded and implemented with a range of affordable agonist medications available.
